# Examining the connection among national tourism expenditure and economic growth in Algeria

**DOI:** 10.1186/s43093-021-00059-8

**Published:** 2021-04-01

**Authors:** Salah Eddine Sari Hassoun, Khayereddine Salim Adda, Asma Hadjira Sebbane

**Affiliations:** 1 LEPPESE Laboratory, Center University of Maghnia, Tlemcen, Algeria; 2 Poldeva Laboratory, Abou Bekr Belkaid University, Tlemcen, Algeria; 3 MIFMA Laboratory, Abou Bekr Belkaid University, Tlemcen, Algeria

**Keywords:** Tourism, Economic growth, Unit root test with and without breakpoint, Cointegration with breakpoint, Breitung and Candelon causality, L83, C19

## Abstract

Tourism is one of the most important sectors for several researchers and decision makers, due to its influence on the world economic growth in the twenty-first century, making it as a source of competition between countries to a global industry for its effective strategic role in the development of countries. In this paper, we used two variables natural logarithm of per capita gross domestic product (GDP) and natural logarithm of per capita international and national tourism expenditure (ITE) to study the relationship between the tourism sector and economic growth in Algeria over the period of 1995–2017. We established with the unit root test with and without breakpoint that the variables are stationary in the first difference and there is a structural break in (ITE) and (GDP). Thus, with the presence of a breakpoint, we employed the methodology of Gregory–Hansen to avoid such issue, but we found that there was no evidence of cointegration with breakpoint, so then we used the vector autoregressive model (VAR). The model showed that the tourism sector has a positive and insignificant coefficient on the economic growth, while the economic growth factor has a positive and significant on the tourism sector. In the short run, there was a one-way causality from GDP to ITE at the level of 1%, confirming the economic-driven tourism growth hypothesis. Also, we found with Breitung and Candelon causality that there was same causality at the level of 10%.

## Introduction

The economic growth has been linked with different sectors such as basic agricultural, tourism and industrial sectors as well as the flow of foreign capital. But, nowadays, many nations should diversify their economic situation and they ought to give more importance to the tourism sector. In 1999, Papatheodorou [41] concluded from his analysis in the Mediterranean region that the role of tourism in economic growth has been underestimated as a sector without a clear growth trend. Since then, the tourism sector has started to take a major place in society and it has drawn attention to its ability to expand and diversify into becoming one of the fastest growing economic sectors in the world. Tourism is growing continuously despite repeated infrequent crises due to terrorism, natural disasters and COVID-19 pandemic. Also, it plays a strategic role in the government development and it represents a vital element in achieving sustainability and increases the investment possibilities. According to the World Tourism Organization, the number of international tourist arrivals^1^ expanded at an annual rate of 6.2% and increased from 25 to 980 million tourists from 1950 to 2011 [26]. According to World Bank Statistics [24],^2^ it shows that the number of international tourist arrivals rose from 524 million in 1995 to more than 1 billion and 341 million in 2017.

Tourism has not only a particular importance regarding to researchers and decision makers, but it is an essential component of the world economy’s todays as well. Besides, it becomes a competitive global industry for its effective strategic role in the development of nations, where a group of researchers has concluded that there is a single trend of economic growth to the tourism sector [2]. Also, there are some scholars who found that the tourism sector impact and cause positively the economic growth [8, 9, 20, 42, 48, 53]. Moreover, the investment in the industry sector is a vital element in achieving sustainability for any country, and any rise in the capital flows will affect the size and the distribution of different tourism projects and the flow of their aggregates to the regions of increasing their revenues and profits [21]. Therefore, the development of the tourism services will be beneficial for any country, where some of them have a modest natural resources and wealth. Thus, with good tourist assets, any country will be able to build a strong economy based on the tourism sector [11, 19], but setting up a country for tourism is difficult due to the cost of the infrastructures.

Algeria, like other countries, seeks to achieve a comprehensive development that reaches the socioeconomic growth and stability, but its economic situation depends mainly on the oil sector. To finance and to diversify the process of the economic development, Algeria should focus on the tourism sector due to the possibilities that can offer. Hence, an adoption of a huge tourism program and strategic investment will generate a high profit, which they will be aimed at promoting and supporting the industrial economy without depending a lot on the oil sector. However, the reality has proved that the measures taken and the limited sector are not effective in contributing to the economic growth and therefore did not rise to an alternative developmental level due to the obstacles that prevented it, such as the Arab Spring, an unprecedented wave of political violence and terrorist acts which have happened in several MENA tourism-dependent economies, keeping tourists away from Egypt, Tunisia and Turkey, and also now the health issue with COVID-19 pandemic which has cut every economic activities around the world. The goal of this study is to investigate the relationship between the tourism sector and economic growth in Algeria during the period of 1995–2017.

**Fig. 1:**
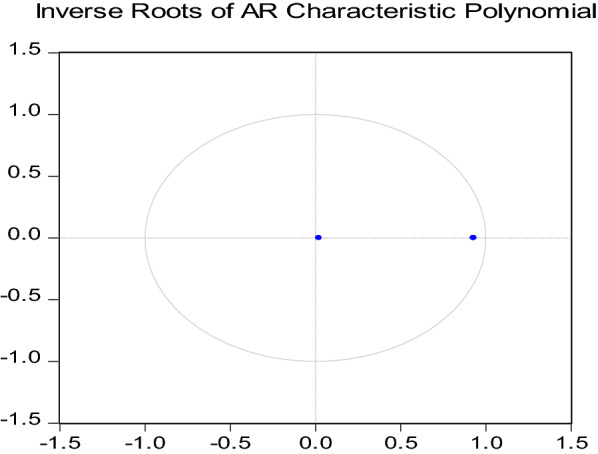
Inverse roots of AR characteristic polynomial. *Source*: Done on EViews 10. This graph showed that the model VAR is stationary, because the two roots lie inside the unit circle (under root 1)

This paper demonstrates the importance and the role that can play the tourism sector in the Algerian economic growth by using an econometric model. This study is divided into five sections, the introduction, the literature review, the data and methodology, the empirical result and conclusion plus reference and “Appendix”.

## Literature review

A large literature has examined the connection amongst tourism and economic growth for several nations, often showing the relationship to vary depending on the specific country investigated, the time periods considered and the methods employed. One strand of literature argues for tourism-led economic growth (TLEG) hypothesis that views tourism as a strategic factor for long-term domestic economic growth, generating direct, indirect, or induced effects on other productive sectors [57]. Balaguer and Cantavella-Jorda [3] found a one-way causality from tourism to economic growth in Spain. Brida et al. [7] reported a positive impact of tourism expenditure on GDP per capita in Uruguay. Dritsakis [17] confirmed the beneficial effect of tourism on GDP in seven Mediterranean countries. Related results are established in Lanza et al. [31] for 13 OECD countries, Durbarry [18] for Mauritius, Gunduz and Hatemi-J [23] for Turkey, Proença and Soukiazis [47] for several southern European countries, Brida and Risso [10] for South Africa, Belloumi [5] for Tunisia and Katircioğlu [27] for Singapore, among others. In fact, of the 87 empirical analyses studied, Pablo-Romero and Molina [40] described that 55 investigations showed evidence in support of the TLEG hypothesis.

Contrary to the TLEG hypothesis, the second stream of literature declares that economic variations are the driving force behind the tourism sector, which is often referred to as the economic-driven tourism growth (EDTG) hypothesis. The reasoning underpinning the EDTG assertion is that resource availability, infrastructure development and political stability creates an ambient economic climate that promotes tourism activities. Oh [38] established that the economic growth Granger-causes tourism in South Korea, but not vice versa in the short run. The studies of [32, 37, 43] confirmed an evidence of the EDTG hypothesis in various other countries.

A third hypothesis, called the feedback or reciprocal hypothesis, claims that there exists a bi-directional feedback connection amongst tourism and economic growth. For instance, Dritsakis [16] analyzed the influence of the tourism sector and real exchange rates on the economic growth in Greece during the period of 1960–2000. The results showed that the three variables are cointegrated and that a bi-directional causal relationship exists between tourism and economic growth. The two-way link among tourism and economic growth was also obtained for Taiwan by Kim et al. [29] and Lee and Chien [32], for Malaysia by Tang [55] and for Spain by Perles-Ribes et al. [44].

However, some scholars have found evidence in support of a fourth hypothesis that no causality exists between tourism and economic growth [28].

The World Economic Forum [25]^3^ in 2018 displayed the importance of the government support for the sustainable travel and tourism all around the world. Lee and Jan [34] saw that the government spending can contribute to the promotion of tourism sector and then to the sustainable development and the economic growth. According the scholars [15, 33, 51, 52] the tourism sector has an important role in enhancing the level of social and economic welfare. Croes [14] have indicated that tourism has the potential for improving quality of life; and a greater understanding of the relationship between human development, economic growth and tourism in the context of developing countries has warranted attention. Rivera [48] examined the relationship between human development, economic growth and tourism sector with cointegration methodology and Granger causality in the case of Ecuador. The results indicated that the tourism arrivals have a positive but insignificant impact on the Human Development Index and there is a unidirectional causality running from HDI to tourism arrivals.

Aratuo and Etienne [1] investigated the link between GDP and the real output of six tourism industries in the USA and within the tourism industries using quarterly data from 1998–2017. They established that there is no cointegration except for the lodging, food and beverage sectors. The Toda–Yamamoto causality shows that there is a one-way causality running from GDP to the six tourism sectors, indicating the economy-driven tourism growth hypothesis predominantly observed in developed countries where tourism revenue only accounts for a small portion of the overall economy. Sokhanvar [54] examined the connection among the foreign direct investment, economic growth and tourism development with the impulse responses. The result showed that the international tourism expansion is of great importance for economic growth in Bulgaria and Spain, which can be the evidence of the significant role of tourism in the improvement of standards of living in these countries. Therefore, economic growth can be stimulated by subsidizing tourism in these countries more than the other countries. Improving tourism offer structure in tourist destinations can enhance the level of tourism receipts in these countries. Roudi et al. [49] did a study about the relationship between tourism and economic growth for the small island developing states during the period of 1995–2014 and he confirmed the positive and significant effect of tourism sector on the economic growth. Ohlan [39] studied the connection amongst the economic growth and tourism sector in India during the period of 1960–2014. He employed the methodology of ARDL and Granger causality to examine the short- and the long-run relationship between GDP, tourism sector revenue and financial growth. He established that the two variables have a positive effect on GDP and there is an evidence of unidirectional causality running from the tourism sector to GDP, confirming the Tourism-Led Economic Growth Hypothesis. Chulaphan and Barahona [12] made a model about the tourism sector and the economic growth in Thailand over the period of January 2008 to November 2015. They used the VAR procedure and Granger causality to analyze the link among the number of tourism arrival and industrial production index as a proxy of economic growth; as result, they concluded that there is a unidirectional causality running from the number of tourism arrival from Southeast Asia to industrial production index, while there was another one-way causality running from the proxy of the economic growth to the number of tourism arrival from Oceania. Cro and Martins [13] employed the procedure of unit root with breakpoint to evaluate the tourism sector on 25 countries during the period of 1995–2014. They found that there is a break in 2003 in Asia, confirming that the natural disaster reduced the number of tourism arrival, especially in Eastern Asia. However, there was another break in 2011 for the MENA country, showing that the terrorism and Arab Spring affected negatively the number of tourism arrival in this region. Seghir et al. [53] analyzed the link between tourism spending and economic growth for 49 countries by using FMOLS, DOLS and panel Granger causality during the period of 1988–2012. They established that an increase in tourism spending will surge the level of the economic growth in these countries; meanwhile, there is a one-way causality running from GDP to tourism spending, confirming the Economic Development Draven Tourism Hypothesis.

## Methods

The oil and natural gas sectors have long been the backbone of the Algerian economy, accounting for roughly 60% of budget revenues, 30% of GDP and over 95% of export earnings. Their exports have enabled the country to maintain macroeconomic stability and accumulate large foreign currency reserves and a large budget stabilization fund available. In addition, Algeria's external debt is extremely low at about 2% of GDP. However, the country is now struggling to develop non-hydrocarbon industries because of its regulations and policies such as tourism sector.

### Data

We shall use two variables to investigate the relationship between tourism sector and economic growth in Algeria during the period of 1995–2017. The variables are the natural logarithm per capita gross domestic production (GDP) and the natural logarithm per capita international tourism expenditures (ITE).

The variables are transformed into natural logarithm specification, because the coefficient on the natural-log scare is directly interpretable as approximate proportional differences and as elasticity. This transformation has provided us with the following benefits, problems related to dynamic qualifications of the data set are avoided log-linear specification, and it gives more consistent and efficient empirical results [50] (Table 1).

**Table 1:** Variables definition

Variables	Unit	Source
GDP	Current US $	World Bank
ITE	Current US $	World Bank

### Model and methodology

In this paper, we will make a univariate analysis about the tourism variable with the test of structural breaks unit root to find whether there is an influence of the crisis or shock on Algerian tourism during the period 1995–2017.

After the major findings of Nelson and Plosser [35], the traditional view of the unit root hypothesis was that the current shocks only have a temporary effect and the long-run movement in the series is unaltered by such shocks. Perron [45] claims that a structural variation and unit roots are closely related, so the scholars ought to bear in mind that the basic unit root tests are biased toward a false unit root null when the data are trend stationary with a structural break. This observation has spurred development of a large literature outlining various unit root tests that remain valid in the presence of a break. “Most macroeconomic time series are not characterized by the presence of a unit root. Fluctuations are indeed stationary around a deterministic trend function. The only ‘shocks’ which have had persistent effects are the 1929 crash and the 1973 oil price shock”.

Zivot and Andrews [59], Banerjee et al. [4], Vogelsang and Perron [58], and Perron [46] claim that the outcomes from the conventional unit root tests (ADF, PP, KPSS and Ng-Perron) may be reversed by endogenously determining the time of structural breaks. They therefore suggested a novel approach to test whether there is a structural change or not, so the null hypothesis states that there is unit root or the unit root with structural breaks, and the alternative hypothesis is a stationary process that allows for a one-time unknown break in intercept and/or slope, using the following regression equations:

The univariate model can be written as follows:

1 $${\Delta }_{yt}=c+{\alpha }_{yt-1}+{\beta }_{t}+{\gamma DU}_{t}+\sum_{j=1}^{k}{d}_{j}{\Delta }_{yt-j}+{\varepsilon }_{t}$$

2 $${\Delta }_{yt}=c+{\alpha }_{yt-1}+{\beta }_{t}+{\theta DT}_{t}+\sum_{j=1}^{k}{d}_{j}{\Delta }_{yt-j}+{\varepsilon }_{t}$$

3 $${\Delta }_{yt}=c+{\alpha }_{yt-1}+{\beta }_{t}+{\gamma DU}_{t}+{\theta DT}_{t}+\sum_{j=1}^{k}{d}_{j}{\Delta }_{yt-j}+{\varepsilon }_{t}.$$

where *DU*_*t*_ is a dummy variable or an indicator for a mean shift occurring at each possible break point, while *DT*_*t*_ is the trend shift variable, as follows:

- *DU*_*t*_ = 1 if *t* > TB and 0 otherwise.
- *DT*_*t*_ = *t*−TB if *t* > TB and 0 otherwise.

In our investigation, we shall write the univariate model as follows:

4 $${ITE}_{t}={\beta }_{j}+{\epsilon }_{t}$$

with $$t={T}_{j-1},\ldots,{T}_{j}.$$

ITE_*t*_: is the variable of the tourism sector over time “t”. It includes the expenditures of international outbound visitors in other countries, including payments to foreign carriers for international transport (World Bank).

With *J* = 1…*m* + 1 and $${\beta }_{j}({J}=1,\ldots{ m}+1)$$

${\beta }_{j}$: is the mean of the endogenous variable on level of «J».

(*T*_*j*_,….,*T*_*m*_): is the breakpoint defined on one or different levels, and it could be one break or several breaks.

After testing the unit root with the breakpoint, we shall investigate the existence of the cointegration with the breakpoint. Gregory–Hansen [22] proposed an opposite view what did Engel–Granger test, Johansen–Juseluis test and bound test (ARDL) addressed the issue of estimating the cointegration connection in the presence of a potential structural break. Kunitomo [30] declared that the presence of a structural change and traditional cointegration tests, which do not allow for this, may produce spurious cointegration; for this reason, Gregory and Hansen proposed the following equations:

5 $${y}_{t}={a}_{0}+{a}_{1}{\theta }_{t}+{a}_{2}{x}_{t}+{\varepsilon }_{t}.$$

6 $${y}_{t}={a}_{0}+{a}_{1}{\theta }_{t}+{a}_{2}{x}_{t}+{a}_{3}t+{\varepsilon }_{t}.$$

7 $${y}_{t}={a}_{0}+{a}_{1}{\theta }_{t}+{a}_{2}{x}_{t}+{a}_{3}{x}_{t}{\theta }_{t,\pi }+{\varepsilon }_{t}.$$

with *t* = 1…*n*.

Where $t,\pi$ is a dummy variable and ${\theta }_{t,\pi }=1$ if $t>n\pi$ or 0 if $t\le n\pi$, and $\pi \in ({0,1})$ denotes the relative timing of the break point, in this model the effect of structural break is only on the intercept, which μ0 is the intercept before the break, and *a*_0_ is the change in intercept at the time of the break.

On the other hand, in this paper, we will use the modern causality of Breitung and Candelon [6]. The authors proposed a modified frequency domain causality using the VAR specification as follows:

8 $${M}_{t}= {\omega }_{1}{M}_{t-1}+\cdots + {\omega }_{p}{M}_{t-p}+\cdots +{\partial }_{1}{N}_{t-1}+\,{\partial }_{p}{N}_{t-p}+{\varnothing }_{t}$$

And the new null hypothesis became *H*_0_: *R* (*ω*) where Ω constitutes a vector of coefficients of *N* and *M*.

$$R\left(\omega \right)= \left[\begin{array}{cc}{\cos}\left(\omega \right)& cos\left(2\omega \right) \cdots {\cos}(p\omega )\\ {\sin}\left(\omega \right)& sin\left(2\omega \right) \cdots {\sin}(p\omega )\end{array}\right].$$

The F-statistic for this equation follows *F* (2, *T* − 2*p*) for *ω* ∊ (0, *π*), and it is necessary to be noted that the high frequencies represented the short-run term causality and the low frequencies represented the long-run term causality, and as considered by Toda and Phillips [56] in cointegration systems the definition of causality of frequency zero is equivalent to the concept of long-run causality.

## Results and discussion

### Unit root tests

We employ two types of unit root tests, the first one without structural breaks [36] and the second one with structural breaks (Zivot-Andrews; Perron). We selected the Schwarz info criterion for the optimal lag length selection, and all tests are run with a constant and trend term to determine the integration degree.

From Tables 2 and 3, we displayed the Ng-Perron test and we concluded that both variables are neither stationary on level, nor stationary on first difference for the model with constant and linear trend. However, for the model with constant only, GDP and ITE are stationary on 1st difference.

From the tests of Zivot-Andrews and Perron, we see that both series are non-stationary on level, while the structural breaks occurred in the period 2004–2005 for the variable of tourism and in the period of 2010, 2011 and 2014 for the variable of the economic growth. Therefore, in 2004–2005, Algeria has known some political and social instability that pushed the country to change its view over the tourism sector in order to enhance and to improve the revenue from this sector. However, in 2010–2014, we can say that the major factor that participates to decrease the level of tourism revenue is the Arab Spring and some terrorist attacks.

The outcomes from unit root test without and with structural break confirm that we need to use the Gregory–Hansen test of cointegration with regime shift (with structural breaks).

### Gregory–Hansen test

Ignoring the issue of potential structural breaks can render invalid the statistical results not only of unit root tests, but also of cointegration [45], also Kunitomo [30] shows that the ignoring of the structural breaks in the procedure of cointegration may produce spurious cointegration, for this reason, we must run the Gregory–Hansen [22] test to examine the long-run relationship with regime shift among the variables.

The outcomes display that there is a no cointegration with the breakpoint in both model 1, 2 and 3. Thus, according to this result, we will make a basic vector autoregressive model to show the short-run relationship between GDP and ITE.

### VAR estimations

The optimal model is with lag 1 (*p* = 1) according to the three criteria (AIC), (SC) and (HQ). We then estimate the VAR model with the optimal lag 1. Also, the Fisher statistic appears very good, so both models are globally significant.

The coefficient of ITE is positive and insignificant for both models, confirming that the tourism sector has not yet a direct influence on the Algerian economic growth; it can be explained by the lack of the infrastructure, luxury hotels and advertising.

However, the sign of GDP is positive and statistically accepted for both models, indicating that the GDP represents a major force behind driving most of the economic infrastructure assets, while this result can show that there is a unidirectional causality running from GDP to ITE.

The autoregressive root graph showed that the model VAR is stationary or stable, because, the two roots lie inside the unit circle.

### Causality results

The short-run Granger causality indicates that there is a one-way relationship from GDP to ITE at the level of 1%, approving that the economic variations are the driving force behind the tourism sector, which is often referred to as the economic-driven tourism growth (EDTG) hypothesis. The reasoning underpinning the EDTG assertion is that resource availability, infrastructure development, and political stability creates an ambient economic climate that promotes tourism activities. This finding is in line with the studies of Oh [38], Lee and Chien [32], Payne and Mervar [43], Odhiambo [37].

In the long run, we shall employ the modern Breitung-Candelon frequency domain causality, the result displays that there is evidence of one-way causality from GDP to ITE at all the frequencies, so there is a causal effect running from the economic growth to the tourism sector at the level of 10%.

## Conclusion

This paper studied the influence of tourism sector on the economic growth in Algeria during the period 1995–2017 with some econometric tools. The literature review described some significant study and indicated that there are many outcomes and hypothesis about the real connection amongst tourism and economic growth.

The unit root test without breakpoint shows that both variables are stationary in the first difference I (1), indicating that we can apply the cointegration procedure for this case, but, confirmed by the unit root test with breakpoint and it displays that both variables have a structural change, so there is some crises or shock that occur in both variables and it may have a serious impact on the selection of cointegration test method.

In order to avoid such issue, Gregory and Hansen proposed a cointegration test with breakpoint methodology. Therefore, they made their program and model on four models, but in this study we tested only 3 models. The findings show that there is no evidence of cointegration with the breakpoint. We thus estimate a classical VAR model to examine the influence of the tourism sector on economic growth and vice versa.

The result of VAR estimation indicates that the model is perfectly defined, while both variables have a positive sign, but significant for GDP and insignificant for ITE. This demonstrates that such country do not a huge importance to the tourism sector to enhance and to develop its economic growth in the present.

The outcomes from causality show that there is evidence of unidirectional causality running from GDP to ITE at the level of 1%, confirming the (EDTG) hypothesis. However, the Breitung and Candelon spectral density causality shows the same causality, but at the level of 10%.

To boost foreign tourism, Algeria must focus on improving the domestic tourism by making efforts on development in air transport and all transport infrastructure and development of mass-market package tourism. However, the Algerian tourism sector’s potential is difficult to dispute, given the country’s natural assets, its large domestic market and the under-developed state of infrastructure.

Therefore, the authorities have the power in their hand to mobilize sustained investment in the current challenging economic environment and ensuring institutions with low-cost options to attract more tourists in Algeria.

## Data Availability

The datasets generated during and/or analyzed during the current study are available in the World Bank. EViews 10 license is obtained from University of Tlemcen. We use demo license of Stata 15.1

## Appendix

See Fig. 1 and Tables 2, 3, 4, 5, 6, 7, 8, 9.

**Table 2:** Unit root test without structural breaks (Ng-Perron) for the model with the constant and linear trend

Variables	MZa	MZt	MSB	MPT
GDP	− 2.698	− 0.905	0.335	25.966
Δ(GDP)	− 10.394	− 2.276	0.219	8.779
ITE	− 9.212	− 2.077	0.225	10.143
Δ(ITE)	− 9.210	− 2.140	0.232	9.915

*Source*: Done on EViews 10

The critical values at 10% are: − 14.2, − 2.62, 0.185 and 6.67

The critical values at 5% are: − 17.3, − 2.91, 0.168 and 5.48

The critical values at 1% are: − 23.8, − 3.42, 0.143 and 4.03

*, **, and *** denote that the series are stationary at 10%, 5% and 1%

**Table 3:** Unit root test without structural breaks (Ng-Perron) for the model with the constant only

Variables	MZa	MZt	MSB	MPT
GDP	− 0.348	− 0.284	0.814	36.113
Δ(GDP)	− 10.171**	− 2.251**	0.221**	2.421**
ITE	− 3.260	− 1.205	0.369	7.425
Δ(ITE)	− 9.295**	− 2.151**	0.231	2.651

*Source*: Done on EViews 10

The critical values at 10% are: − 5.7, − 1.62, 0.275 and 4.45

The critical values at 5% are: − 8.1, − 1.98, 0.233 and 3.17

The critical values at 1% are: − 13.8, − 2.58, 0.174 and 1.78

*, ** and *** denote that the series are stationary at 10%, 5% and 1%

**Table 4:** Unit root test with structural breaks

Variables	ZA statistic	Break	1%	5%	10%	Decision
Zivot-Andrews test						
GDP	− 2.808	2011	− 5.57	− 5.08	− 4.82	I(1)
ITE	− 5.873***	2005	− 5.57	− 5.08	− 4.82	I(1)

Variables	Innovative outliers		Additive outliers		Decision	
	*t* statistic	Break	*t* statistic	Break		
Perron test						
GDP	− 3.843	2014	− 3.851	2010	I(1)	
ITE	− 5.155**	2004	− 6.303***	2004	I(1)	

*Source*: Done on EViews 10

*, ** and *** denote that the series are stationary at 10%, 5% and 1%

**Table 5:** Gregory–Hansen test

Test	Test statistic	Breakpoint	Asymptotic critical values		
			1%	5%	10%
Model 1: break in the constant					
ADF	− 4.15	2009	− 5.13	− 4.61	− 4.34
Zt	− 4.25	2011	− 5.13	− 4.61	− 4.34
Zα	− 20.64	2011	− 50.07	− 40.48	− 36.19
Model 2: break in the constant and trend					
ADF	− 3.40	2013	− 5.45	− 4.99	− 4.72
Zt	− 3.48	2013	− 5.45	− 4.99	− 4.72
Zα	− 16.76	2013	− 57.28	− 47.96	− 43.22
Model 3: break in the constant and slope					
ADF	− 4.44	2006	− 5.47	− 4.95	− 4.68
Zt	− 4.54	2006	− 5.47	− 4.95	− 4.68
Zα	− 22.12	2006	− 57.17	− 47.04	− 41.85

*Source*: Done on Stata 15.1

**Table 6:** VAR lag order selection criteria

Lag	AIC	SC	HQ
0	0.812	0.911	0.829
1	− 1.260*	− 0.962*	− 1.209*
2	− 1.069	− 0.572	− 0.985
3	− 0.790	− 0.094	− 0.673
4	− 0.604	0.290	− 0.452

*Source*: Done on EViews 10

* indicates the optimal model

**Table 7:** VAR estimation

	ITE	GDP
ITE_*t*−1_	0.145	0.150
*t*-student	0.587	1.106
GDP_*t*−1_	0.645***	0.804***
*t*-student	2.884	6.523
C	− 3.043**	1.237*
*t*-student	− 2.380	1.755
*R*^2^	0.756	0.937
*F*-statistic	29.570***	143.331***

*Source*: Done on EViews 10

*, **, ***, denotes the student table for *n* = 22 (10% = 1.717), (5% = 2.074) and (1% = 2.819)

*** indicates the Fisher table for *v*_1_ = 1 and *n* = 22 (1% = 7.94)

**Table 8:** Granger causality in the short term

	*χ*^2^	Prob
Dependent variable: ITE		
GDP	8.318	0.0039
Dependent variable: GDP		
ITE	1.223	0.2686

*Source*: Done on EViews 10

**Table 9:** Breitung and Candelon spectral density causality (*p* = 2)

Frequencies	*ω* = 0.00	*ω* = 1.00	*ω* = 2.00	*ω* = 3.00	Critical value
GDP ≥ ITE	5.442*	5.442*	5.442*	5.442*	6.00
*p* value	0.0658	0.0658	0.0658	0.0658	
ITE ≥ GDP	2.992	2.992	2.992	2.992	6.00
*p* value	0.224	0.224	0.224	0.244	

*Source*: Done on Stata 15.1

## Abbreviations

GDP: Natural logarithm of per capita gross domestic product
ITE: Natural logarithm of per capita international and national tourism expenditure
VAR: Vector autoregressive model
ARDL: Autoregressive distributed lag model
AIC: Akaike information criterion
SC: Schwarz information criterion
HQ: Hannan–Quinn information criterion

## Footnotes

1 Represents the most common unit of measure used to quantify the volume of international tourism, and it refers exclusively to tourists or overnight visitors.

2 https://data.worldbank.org/indicator/ST.INT.ARVL.

3 https://www.weforum.org/.
